# Expanding the cryogenic electron microscopy toolbox to reveal diverse classes of battery solid electrolyte interphase

**DOI:** 10.1016/j.isci.2022.105689

**Published:** 2022-11-30

**Authors:** Elizabeth Zhang, Matthew Mecklenburg, Xintong Yuan, Chongzhen Wang, Bo Liu, Yuzhang Li

**Affiliations:** 1 Department of Chemical and Biomolecular Engineering, University of California Los Angeles, Los Angeles, CA 90095, USA; 2 California NanoSystems Institute (CNSI), University of California, Los Angeles, California 90095, USA

**Keywords:** Materials characterization, materials chemistry, materials science

## Abstract

Advancements in energy storage technologies such as Li-based batteries depend on a deep understanding of the chemical and structural aspects of critical interfaces. Among these, the solid electrolyte interphase (SEI) governs how batteries operate yet remains one of the most elusive to characterize due to its rapid degradation under an electron beam and sensitivity to ambient conditions. In recent years, cryogenic electron microscopy (cryo-EM) has emerged as a promising technique to provide atomic-resolution imaging of beam-sensitive battery materials. Distinct SEIs have been discovered with unique chemical compositions and structural features. In this perspective, the role of cryo-EM in uncovering the physicochemical properties of three classes of SEIs (i.e., compact, extended, and indirect SEI) will be defined and discussed. Furthermore, an in-depth analysis of new cryo-EM imaging modalities will be provided to highlight directions for the future development of cryo-EM.

## Graphical abstract

**Figure:**
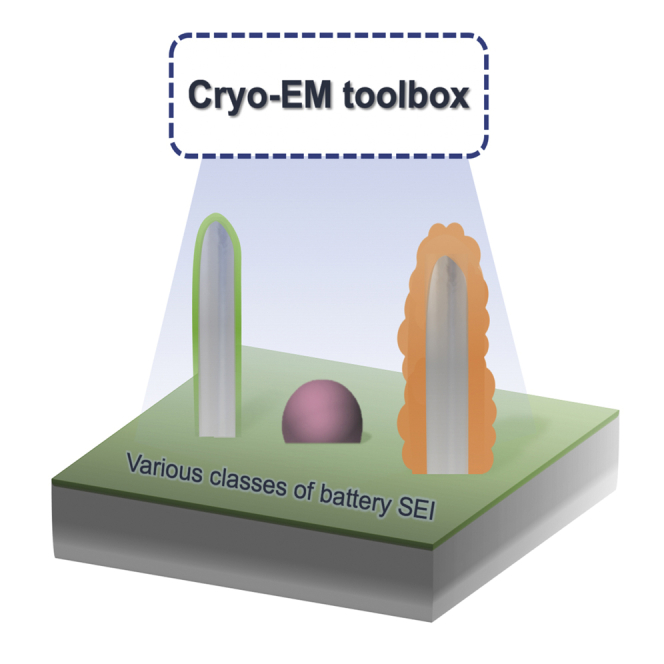

## Introduction

As the world transitions away from fossil fuel energy, the demand for rechargeable battery systems has seen exponential growth.^1^ In particular, lithium-based (Li-based) rechargeable batteries dominate the portable electronics and electric vehicles market. Tremendous research efforts are being invested in the development of battery technologies with higher energy density and cycling stability. However, one of the major obstacles hindering the development of Li-based batteries is our insufficient understanding of the solid electrolyte interface (SEI), which forms on the surface of the anode material (e.g., graphite, silicon, Li metal) as a result of electrochemical reduction of the electrolyte.^2^ Understanding the formation of the SEI layer is fundamental to the design and function of high-performance battery systems, as it directly governs cycling performance and service life of the battery. The concept of SEI was first introduced by Peled et al. in 1979.^3^ Prior to the publication of this theory, electron transfer in batteries was primarily modeled by the Butler-Volmer equation based on the assumption that the rate-determining step is the transfer of electrons from the metal to the cations in the solution.^4^ However, this model was insufficient to explain the formation of a passivating layer observed on the anode. The SEI model proposed by Peled led to our current understanding that SEI primarily consists of insoluble and partially soluble reduction products of the electrolyte components, the result of which is an electronically insulating and ionically conducting self-passivation layer. While the electrically insulating nature of SEI can restrict electrolyte reduction, continuous growth of SEI during cycling consumes active lithium and electrolyte, leading to capacity fade and poor performance.^2^^,^^5^ Although there has been progressing toward the development of “ideal” SEIs (e.g., fully self-passivating), the underlying mechanism of SEI morphological and chemical evolution in various battery systems and chemical environments remains unclear due to its complexity and lack direct measurements of its physical properties.^6^

Past knowledge of SEI primarily relied on ensemble-averaged techniques such as X-ray photoelectron spectroscopy (XPS), X-ray diffraction (XRD), Fourier transform infrared spectroscopy (FTIR), and so forth. While these techniques allowed researchers to investigate the properties of SEI to a certain extent and have greatly advanced our current understanding of SEI up to this point, they each have their own limitations and struggle to provide a comprehensive picture of the SEI. For instance, microscopy and diffraction approaches such as scanning electron microscopy (SEM) and XRD can characterize the SEI by providing insights into its morphology and crystallinity. SEM is arguably the most widely employed technique for the direct visualization of the SEI morphology, cross-sectional views, and elemental analysis over a large area of interest when equipped with energy-dispersive X-ray spectroscopy (EDS). XRD has also been recognized as a powerful technique for obtaining detailed information regarding the crystal structure and phase transformation of the crystalline electrode materials.^7^ However, it is difficult to gather direct information on the chemical bonding environment.^8^ XPS, a commonly employed surface characterization technique, can allow the measurement of surface elemental composition and provides information on the chemical species present within the SEI down to nanoscale spatial resolution in-plane.^9^ Its high sensitivity has allowed monitoring of SEI components such as LiF, Li_2_CO_3_, Li_3_N, and organics.^10^ XPS has thus been widely adopted as a critical technique for understanding the interfacial degradation mechanism in lithium-based battery systems.^8^

However, XPS is limited because the SEI is an inherently poor electronic conductor, posing challenges to phase identification as charging during standard XPS measurements can lead to binding energy shifts that are difficult to correct.^8^ This is further complicated by the presence of multiple Li-containing phases or multilayer structures in the SEI. While spectroscopic methods are usually employed to identify inorganic phases of an SEI, organic phases are more difficult to discern with these techniques. In this regard, FTIR is commonly used to evaluate the organic SEI since most of the salts and carbonate solvents found in electrolytes exhibit IR-active vibrational modes.^11^ The frequency corresponding to the carbonyl stretch, for instance, is highly sensitive to changes in its chemical structures. Coordination with Li^+^ cations can thus be easily detected, yielding information about the carbonyl-based species in the SEI.^12^ Nevertheless, it is challenging to conduct a comprehensive analysis of the SEI since not all components in the SEI are infrared-active.^13^ Nuclear magnetic resonance (NMR) spectroscopy has also been explored to capture the chemical changes that occur at the electrode and electrolyte interface by collecting ^1^H, ^13^C, ^19^F, and ^31^P signals on a timescale. This allows for the detection of SEI components such as LiF (^19^F), Li_2_CO_3_ (^13^C), and other organic components.^10^ Yet, like any other characterization techniques, NMR suffers from issues with sensitivity and selectivity.^14^ Overall, while the current SEI characterization techniques are able to provide a spectrum of information about the interface, they are still insufficient to obtain a local picture of the SEI. Indeed, it is the local chemistry and structure of the SEI which governs heterogeneity and non-uniformity during battery performance and requires high spatial resolution to characterize.

To address the above-mentioned concerns, cryogenic electron microscopy (cryo-EM) has recently emerged as a promising high-resolution imaging technique to capture materials with high beam sensitivity. Awarded the 2017 Nobel Prize in Chemistry for its transformative impact on structural biology, cryo-EM and its adoption toward battery research have significantly advanced our understanding of the nanoscale properties of SEI. For instance, cryo-EM has successfully identified and directly imaged previously proposed models of the SEI (i.e., mosaic and multilayered SEI structure.^15^ Other key findings enabled by cryo-EM include the role of electrolyte additives as well as the effect of temperature^16^ and current density^17^ in various electrolyte systems. These capabilities have been summarized by previous reviews published in recent years.^18^^,^^19^^,^^20^^,^^21^^,^^22^^,^^23^ In this perspective, we highlight and define three classes of SEI structures (i.e., compact, extended, and indirect SEI) that have been revealed through cryo-EM studies, which have led to recent breakthroughs in our understanding of SEI. Note that while cryo-EM has also been applied to study SEI formed in sodium (Na)^24^ and potassium (K)^25^-based battery systems, this perspective will focus on lithium-based systems. Overall, this perspective aims to (1) summarize this growing and evolving knowledge of the critical SEI layer and (2) highlight new imaging modalities that can address remaining unanswered questions within battery research.

### The solid electrolyte interphase: 3 broad classes of solid electrolyte interphase

• **Compact SEI:** A corrosion film ∼5-30 nm in thickness directly interfaced with the anode material.
• **Extended SEI:** A corrosion product on the scale of ∼100 nm that extends out into the electrolyte and is comprised primarily of organic components.
• **Indirect SEI:** In contrast to the compact and extended SEI that is interfaced directly on an anode surface, an indirect SEI describes components that are delocalized from the anode surface.

**Figure 1:**
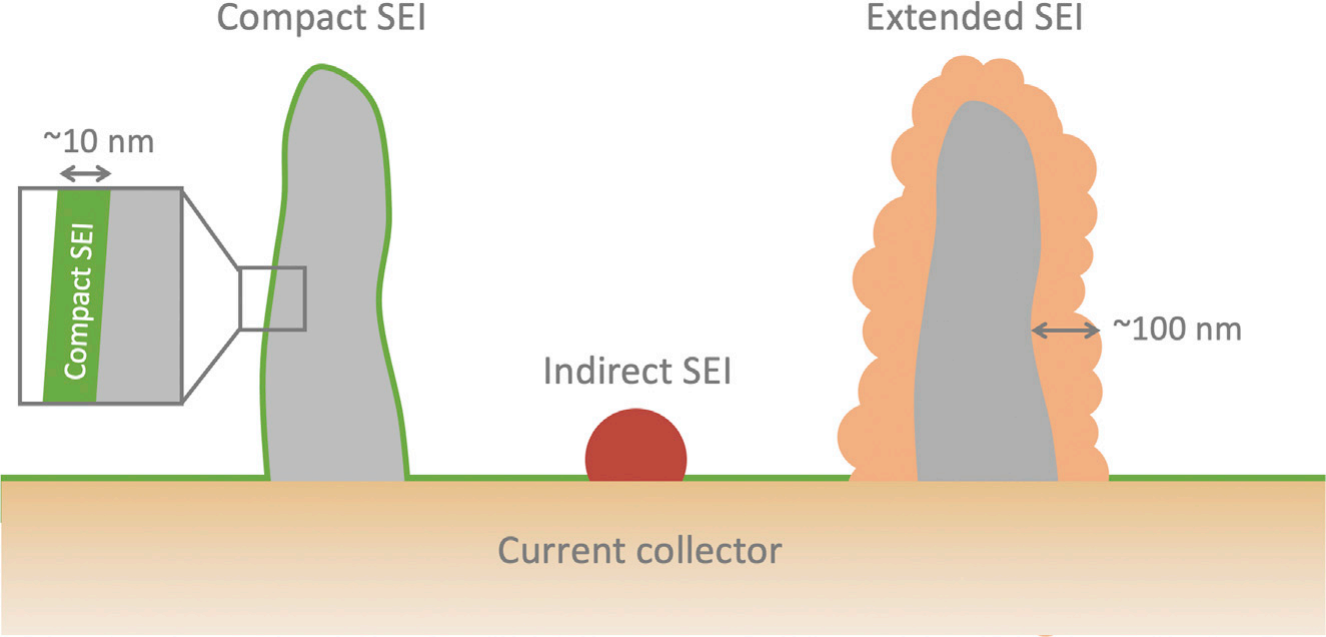
Schematic showing the differences between compact SEI, extended SEI, and indirect SEI in terms of their morphology and length scale

Figure 1 shows a simple schematic of the different types of SEI that may form during battery operation. The descriptions above show that the three broad classes of SEI primarily differ in their length scales and chemical composition. Cryo-EM is thus used to gather these critical information about the samples to distinguish between different types of SEI. The SEI can be further distinguished from the electrolyte with chemical spectroscopy techniques. The following sections will focus on the discussion of each type of SEI, highlighting the contributions of cryo-EM toward enhancing our understanding of its formation and composition.

### Compact solid electrolyte interphase

Since its first introduction in 1979, the understanding of the SEI layer has grown and evolved to encompass some key findings elucidating its origin and growth. The emergence of cryo-EM as a novel SEI characterization technique has brought the understanding of SEI to new heights by allowing the direct identification of various SEI structures with different chemistries and compositions. Thus far, the majority of SEI studies report on the compact SEI, which we define as the 5-30 nm film directly interfaced with the active electrode material and consists of inorganic and organic components.^26^^,^^27^

With innovations in cryo-EM, researchers have been able to unravel previously unknown aspects of this compact SEI layer, three of which we discuss in this perspective:

1. Corroborating the mosaic and multiplayer model previously proposed by Peled et al. and Aurbach et al.
2. Elucidating the role of various electrolyte additives on compact SEI
3. Providing insights into temperature and current density effects on compact SEI formation

Two well-known SEI models have been developed prior to the implementation of cryo-EM technologies, with indirect information determined from ensemble-averaged techniques (e.g., XPS, FTIR). The first is a mosaic structure model proposed by Peled et al., which suggested that the anode surfaces (e.g., Li metal) form a mosaic SEI nanostructure that is composed of inorganic decomposition products heterogeneously dispersed within an organic matrix. Thus, the mosaic SEI is composed of microphases with both amorphous and crystalline attributes.^28^

Alternatively, the multilayer SEI model proposed by Aurbach et al. suggests that the decomposition products in the SEI are uniformly arranged to yield a layered structure with distinct organic and inorganic layers.^28^

**Figure 2:**
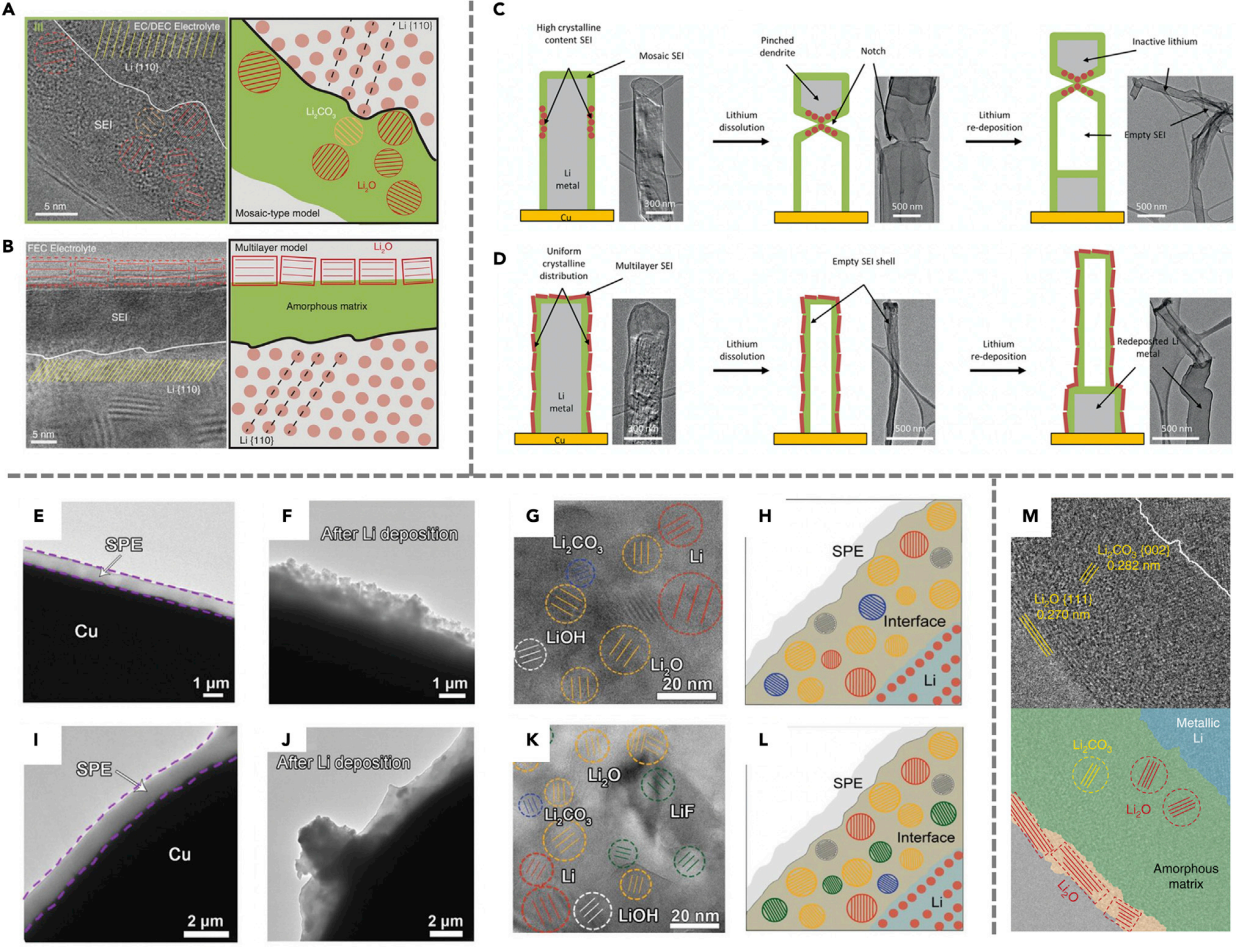
Compact SEI demonstrated using cryo-EM at atomic resolution (A) Atomic-resolution image of the interface between Li metal and SEI, and schematic showing the mosaic model. (B) Atomic-resolution image of SEI formed in FEC electrolyte and schematic showing the multilayered model. (C) Cryo-EM images and schematics of Li metal dendrite formed in EC:DEC, showing inactive lithium formation due to the growth of the notched structure. (D) Cryo-EM images and schematics of Lli metal formed in EC:DEC with 10 vol% FEC additive showing uniformly distributed multiplayer SEI. (E and F) Cryo-TEM characterization of the SEI formed at Li/PEO interface. (G and H) The crystalline grains and schematic diagram for the mosaic SEI structure. (I and J) Cryo-TEM characterization of the SEI formed using PEO-LiTFSI-Li_2_S electrolyte. (K and L) The crystalline grains and schematic diagram for the mosaic SEI structure of SEI formed using PEO-LiTFSI-Li_2_S electrolyte. (M) Cryo-EM image and schematic showing the bilayer structured SEI with the addition of nitrate. Panels (A and B) are adapted/reproduced from ref. ^15^, American Association for the Advancement of Science. Panels (C and D) are adapted/reproduced from ref. ^28^, Elsevier. Panels (E-L) are adapted/reproduced from ref. ^29^, Wiley. Panel (E) is adapted/reproduced from ref. ^30^, Springer Nature Limited.

With cryo-EM, it is now possible to directly image and corroborate these two SEI models to establish the relationship between SEI nanostructure and the various failure modes in Li metal batteries. Cryo-EM has revealed that both models are valid and that the SEI nanostructure is mainly dependent on electrolyte chemistry. Figures 2A and 2B show the formation of a mosaic SEI in ethylene carbonate (EC) and diethyl carbonate (DEC)-containing electrolyte, following the model proposed by Peled et al.^15^ When 10 vol % fluoroethylene carbonate (FEC) is added, however, the Li metal anode surface forms a multilayer SEI nanostructure as proposed by Aurbach et al.^28^^,^^31^ These subtle differences in SEI nanostructure lead to dramatically different impacts on battery performance. During discharging (i.e., stripping of Li metal), cryo-EM observations show that mosaic SEI films lead to the formation of notched Li metal structures, which may contribute to the formation of dead Li due to sections of Li metal that may have been electrically disconnected from the rest of the dendrite (a “pinching off” effect).^28^ Cryo-EM images shown in Figures 2C and 2D indicate that the SEI nanostructure at the notched region features a high concentration of crystalline grains, whereas the nanostructure away from the notch is primarily amorphous, suggesting that the Li-ion transport is non-uniform across the heterogeneous mosaic SEI. It is hypothesized that nanocrystalline components in the SEI can promote Li^+^ transport through the amorphous matrix. The non-uniform spatial distribution of inorganic nanocrystalline domains thus explains the notch formation along the dendrites. In contrast, Li metal exhibiting the multilayer SEI does not exhibit any notched regions during discharge (i.e., stripping of Li metal). As shown in Figure 2D, the cryo-EM images of multilayer SEI show more uniformly dispersed crystalline grains on top of the amorphous matrix. Since no significant increase in the crystalline grain density is observed within the SEI, more uniform Li^+^ transport can be enabled by such multilayer SEI. Based on these observations, it can be inferred that mosaic SEI nanostructure with heterogeneous spatial distribution may result in localized Li dissolution and contribute to the poor cycling performance of batteries without FEC electrolyte additives. These observations enabled by cryo-EM not only offer direct visualization of the SEI nanostructures but also provide critical insights into the mechanism of electrolyte additives.

The impact of other additives such as Li_2_S and LiF on the compact SEI nanostructure is further investigated in a great number of studies. For instance, a recent study explores the interfacial instabilities of solid-state batteries with Li_2_S additives using cryo-EM techniques.^29^ As demonstrated in Figures 2E-2L, the regular Li/polyethylene oxide (PEO) interface consists of large irregular bulk Li deposits. PEO is a polymeric material commonly used as electrolytes for solid-state batteries due to its strong electron-donating ether oxygen groups, excellent thermal stability, and mechanical properties.^32^ In solid-state batteries based on PEO electrolytes, the reactions between Li and PEO are found to result in a heterogeneously porous SPE structure. Unlike in liquid electrolytes, where single-crystalline Li is generally detected, Li exists in the form of polycrystalline particles as it approaches the PEO layer.^29^ Further enlarging the Li/PEO interface, both crystalline and amorphous areas composed of Li metal, LiOH, Li_2_O, and Li_2_CO_3_ can be observed. Upon the addition of Li_2_S, the Li metal is observed to grow into bulk coated with SPE. One significant difference between the system with and without Li_2_S is the presence of a LiF-rich interface composed of uniformly distributed LiF nanocrystals. Such an interface is expected to enhance the diffusion properties and mitigate undesirable reactions between Li metal and PEO.

The incorporation of nitrate anion (NO_3_^−^) in carbonate electrolytes has been extensively studied to elucidate its effects on SEI properties.^30^ More specifically, cryo-EM images revealed that the SEI formed upon the addition of NO_3_^−^ exhibits a bilayer structure as opposed to the mosaic phase that is observed in pristine EC/DEC electrolytes. Interestingly, this SEI morphology appears to influence interfacial chemistry. Unlike the dendritic Li that forms in most carbonate electrolytes, spherical Li nuclei are formed in this case. As shown in Figure 2M, the outer layer of the structure consists of ordered crystalline Li_2_O while the inner phase is primarily composed of Li_2_O and Li_2_CO_3_, with a trace amount of NO_3_^−^ due to the limited concentration of additive added. To further understand the underlying mechanism behind this observed morphology, it is hypothesized that the bilayer structure forms due to the preferential reduction of NO_3_^−^ into insoluble N_x_O_y_^−^ and Li_2_O species, the former of which remains amorphous while the latter forms a dense crystalline layer driven by the kinetically favorable crystallization of Li_2_O. The dense inorganic SEI layer formed as the outer shell promotes uniform Li-ion flux and prevents corrosion of the Li surface. Overall, it is possible that the preferential reduction of NO_3_^−^ can alter the morphology of resulting SEI and further influence the Li deposition.

It is apparent from the abundance of cryo-EM-enabled SEI studies that electrolyte additives play a critical role in the formation of compact SEI. Based on the results from the current studies, one conclusion that can be drawn is that different electrolyte salts and additives with dissimilar electrochemical stability windows decompose to form distinct SEIs. However, a systematic understanding of the underlying mechanisms is still lacking, making it difficult to predict the kind of SEI that will form given an electrolyte system. This may be a fruitful direction for future research efforts.

**Figure 3:**
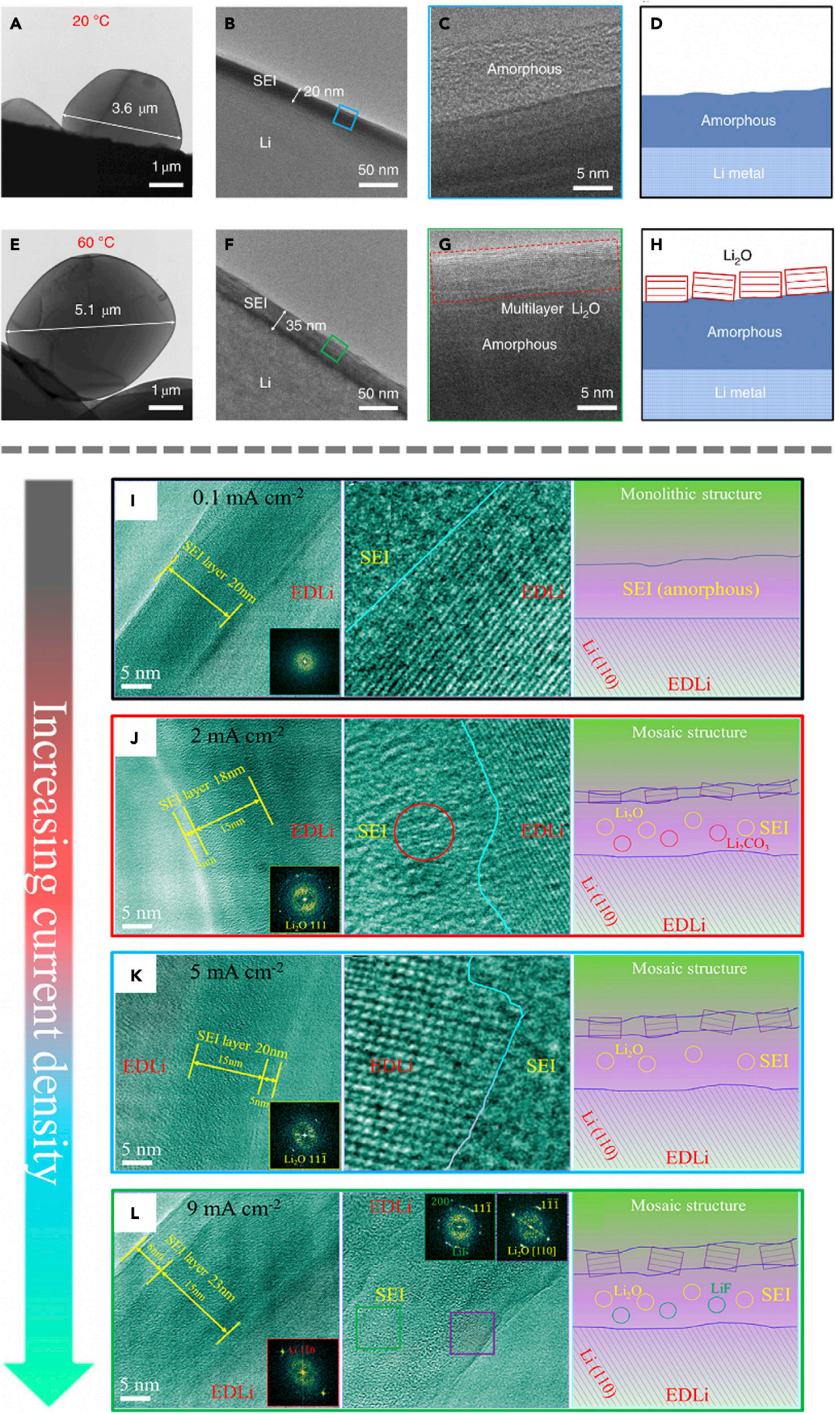
Temperature and current density dependence of compact SEI (A-D) Cryo-EM images and schematics of SEI formed at 20°C. (E-H) SEI formed at 60°C. Layered SEI nanostructure can be observed under higher temperature. (I-L) Cryo-TEM images and corresponding FFT patterns of the SEI and schematics as a function of current density, ranging from 0.1 mA cm^−2^ to 9 mA cm^−2^. Panels (A-H) are adapted/reproduced from ref. ^16^, Springer Nature Limited. Panels (I-L) are adapted/reproduced from ref. ^33^, American Chemical Society.

In addition to structural changes due to additive incorporation, it is also important to point out the effect of temperature and current density on compact SEI formation. In a recent study by Cui et al., cryo-EM revealed that at an elevated temperature of 60°C, a thicker and highly ordered SEI layer (35 nm) is formed on the electrode as compared to the 20 nm formed at 20°C.^16^ The cryo-EM images of the different structures are shown in Figures 3A-3H, and this behavior was attributed to the enhanced reaction kinetics and smaller overpotential at higher temperatures. In addition to the difference in SEI thickness, they also exhibit different nanostructures—the SEI formed at 20°C consists of amorphous polymeric interphase, whereas a layered nanostructure is observed with the SEI formed at higher temperature. This layered nanostructure is composed of an amorphous polymer inner layer and 10 nm of large grains of crystalline Li_2_O directly interfacing with the liquid electrolyte. The nuclei size of Li also increases at elevated temperature, possibly due to the decreased polarization and overpotential, since both the nucleation overpotential and plateau overpotential are observed to decrease with increasing deposition temperature. Further investigating the improved electrochemical performance at elevated temperatures through EIS studies, lower resistance achieved at higher temperatures can be explained by decreased electrolyte viscosity, which allows for enhanced electrode/electrolyte contact, weaker polarization, and lower ion transfer energy barriers. These observations provide critical insights into cycling stability at higher temperatures. More specifically, the amorphous polymeric SEI formed at low temperature cannot provide sufficient passivation of the Li metal and leads to continuous SEI growth since it is soluble in the electrolyte. The layered SEI formed at high temperature, however, is mechanically robust and passivates the anode, leading to enhanced cycling stability. Note that this behavior is not universal across all electrolytes—in electrolyte systems such as EC/DEC, an adverse effect is observed. Although the mechanism entailing how exactly the SEI structures evolve with different electrolyte systems at high temperatures is still unclear, cryo-EM continues to provide insights into the kinetics of SEI formation.

Aside from temperature, the current density is also proposed to have an effect on the composition and morphology of SEI. Recently, Wang et al. reported that at a low current density of 0.1 mA cm^−2^, the SEI layer is monolithic amorphous as observed using cryo-EM.^33^ This is most likely due to the preferential formation of oligomers and polymers at low reaction rates. Increasing the current density to above 2 mA cm^−2^ leads to a thicker SEI with a mosaic structure featuring Li_2_O and Li_2_CO_3_ particles dispersed within the matrix (Figure 3I-L). The outer surface is covered by a thin (3 nm) layer of crystalline Li_2_O. The thickness of this layer increases to 8 nm when the current density is increased to 9 mA cm^−2^, leading to higher impedance. Interestingly, LiF nanoparticles are observed only at high current densities. One possible explanation for this phenomenon is that the Joule heating effect generated by the increased current density causes a small amount of the LiPF_6_ salt to decompose, forming LiF species. The self-heating behavior also results in increased surface migration of Li, thus influencing the SEI formation process.

**Figure 4:**
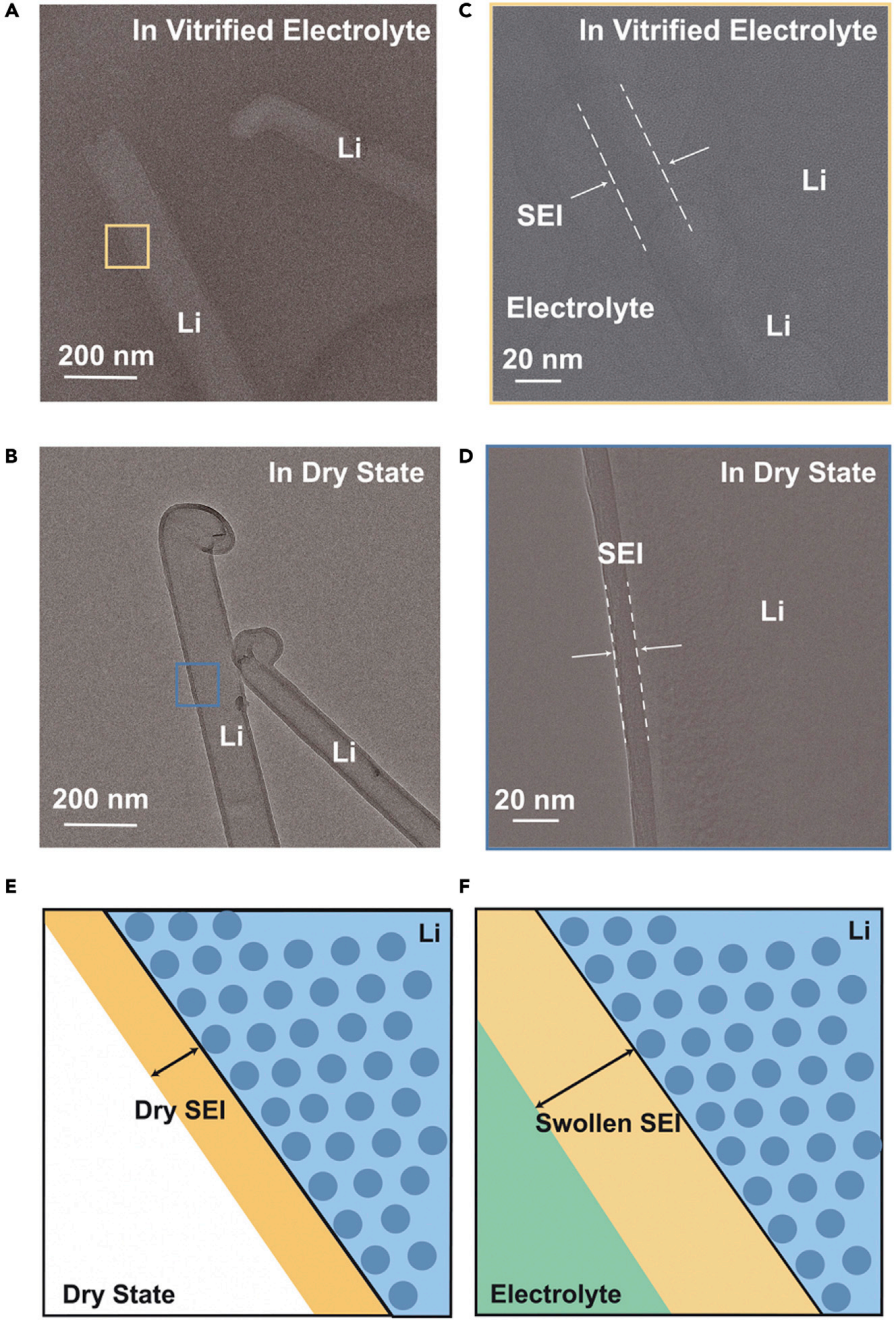
Swelling behavior of SEI captured under cryo-EM (A and B) Cryo-TEM images of SEI on Li dendrite in vitrified organic electrolyte and dry state. (C and D) HRTEM images of SEI on Li dendrite in vitrified organic electrolyte and dry state. (E) Schematic showing the dry SEI under the dry state. (F) Schematic showing the swollen SEI upon the addition of electrolyte. Adapted/reproduced from ref. ^34^, American Association for the Advancement of Science.

Most of these studies on the nanoscale structure of SEI were conducted in the absence of liquid electrolytes. However, it is important to visualize the SEI in the wet state (i.e., when SEI is in contact with liquid electrolyte), as it is a more accurate reflection of real battery operation. A recent study carried out by Cui et al. discovered a swelling behavior of SEI in the liquid electrolyte environment.^34^ As shown in Figure 4, cryo-EM revealed that the thickness of SEI in absence of liquid electrolyte is about 10 nm, whereas the SEI in vitrified/wet state is roughly 20 nm. To further prove the swelling behavior observed under cryo-EM, electron energy loss spectroscopy (EELS) is carried out on the samples. Considering that the SEI is usually composed of both organic and inorganic phases, it is sometimes challenging to discern the organic phases formed from solvent reduction or reactivity with Li. In this regard, EELS core loss and low loss can be used to classify the amorphous species. For instance, oxygen and carbon K-edge spectra can be used to compare the detailed peaks to a reliable reference standard for different species. In this case, EELS revealed that more carbonate-based organic species are present in the wetted SEI. A decrease in elastic modulus further supports the swelling behavior of SEI. Similar behavior is observed in a number of other electrolyte systems, including 1 M LiPF_6_ in EC/DEC with 10% fluoroethylene carbonate (EC/DEC, 10% FEC), 1 M lithium bis(fluorosulfonyl)imide (LiFSI) in 1,2-dimethoxyethane (DME), 4 M LiFSI in DME, and 1 M LiFSI in fluorinated 1,4-dimethoxylbutane (FDMB). These observations are significant as they imply that SEI may not be composed of a dense layer but has a nontrivial amount of electrolyte. Note that similar swelling behavior has also been observed in a CuO nanowire system, where SEI thickens as the potential of the electrode reduces to below 0.0 V.^31^ Thickening of the SEI at this potential indicates that electrolyte penetrated through the SEI and continued to react with Li. Solvent diffusion may therefore be realized as a more significant factor that contributes to the continuous growth of SEI.

**Figure 5:**
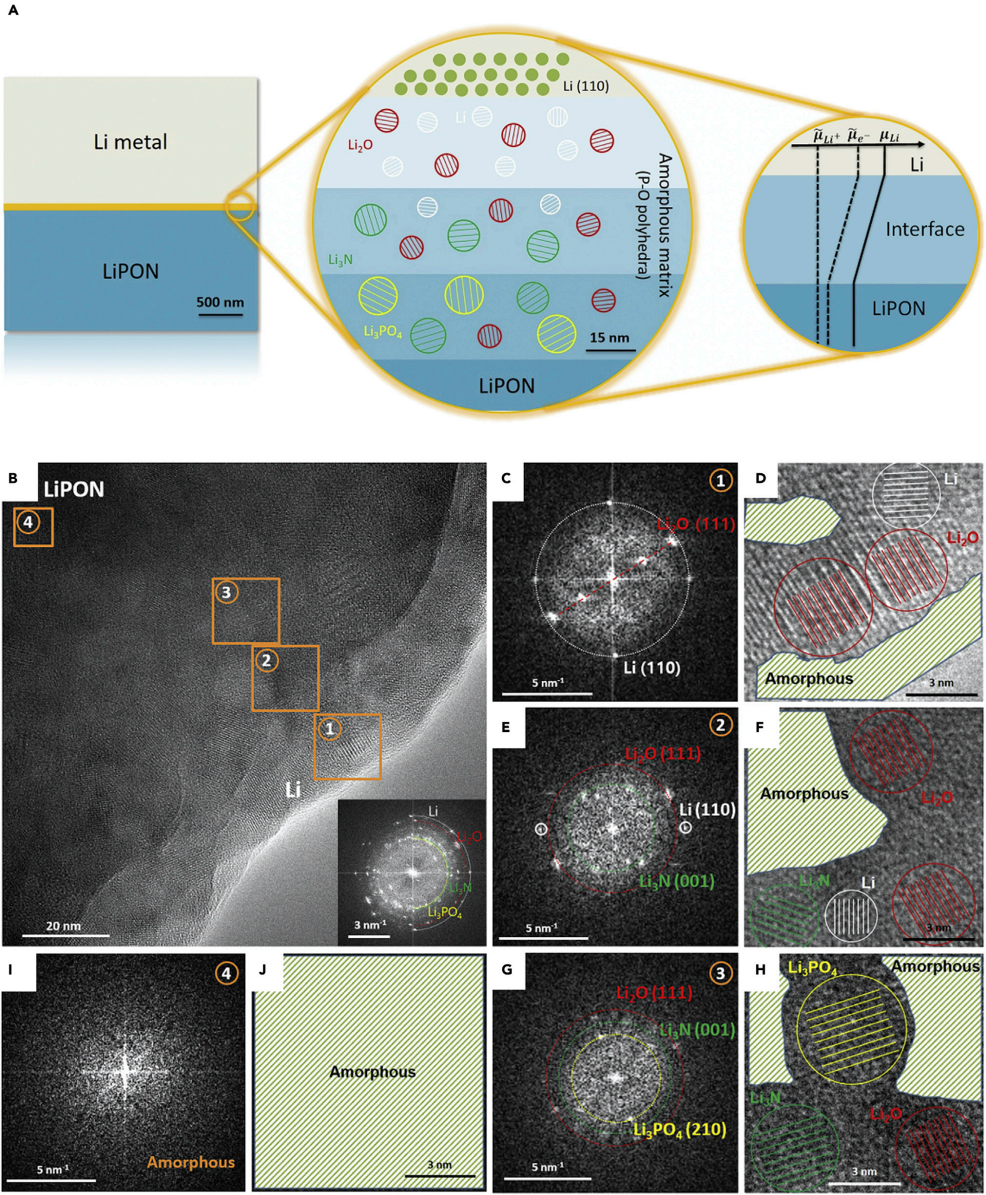
Compact SEI in solid-state battery systems (A) Schematic showing the Li/LiPON multilayered SEI structure, where ${\tilde{\mu }}_{{Li}^{+}},{\tilde{\mu }}_{e^{-}},\mu_{Li}$ are the electrochemical potential of lithium ion, the electrochemical potential of electron, and the chemical potential of lithium, respectively. (B) HRTEM images of SEI divided into four regions as different stages of the multilayered structure. (C, E, I, and G) FFT patterns correspond to the four regions. (D, F, H, and J) Schematic overlaying the HRTEM images corresponding to the four regions. Adapted/reproduced from ref. ^38^, Elsevier.

It is important to note that cryo-EM can also be employed to characterize SEI formed in the all-solid-state battery system. Solid-state electrolytes have drawn considerable interest from researchers lately due to their mechanical strength for dendrite suppression, intrinsic safety, as well as relatively broad electrochemical stability windows.^35^^,^^36^^,^^37^ In this regard, understanding the formation of SEI in these systems is critical to the future optimization of their performance. In a recent study by Meng et al., the interphase between Li metal and lithium phosphorus oxynitride (LiPON), a type of thin film solid electrolyte material, is closely investigated using cryo-EM.^38^ Several observations could be made based on the high-resolution imaging of the interphase. It is found that the SEI consists of an 80nm-thick amorphous multilayer matrix with concentration gradients of nitrogen (N) and phosphorus (P). As shown in the schematic diagram in Figure 5A and the HRTEM images in Figures 5B-5J, the decomposition products such as Li_2_O, Li_3_N, and Li_3_PO_4_ form equilibrium phases that result in an ionically conductive and electronically insulating SEI later. Note that when electronic conductors are also present as decomposition products, the passivating effect of the SEI will be significantly reduced. This work not only highlights the capability of cryo-EM for analyzing a variety of interfaces but also paves the way for future studies on all-solid-state battery interfacial stability.

Overall, these results obtained by using cryo-EM all point to the fact that the structure and chemistry of the compact SEI are inextricably linked with the electrolyte chemistry and operational conditions such as temperature and current density.

### Extended solid electrolyte interphase

Before the development of cryo-EM techniques for high-resolution SEI imaging, our understanding of SEI has been largely limited to the concept of a compact SEI. However, as high-quality chemical and structural mapping is enabled by cryo-EM, multiple studies have demonstrated the presence of a class of SEI known as the extended SEI layer. Unlike the compact SEI, extended SEI is mainly organic in nature and is generally on the scale of hundreds of nanometers that extend out into the electrolyte. Extended SEI is critical to the battery’s performance as such large-scale SEI growth greatly contributes to the Li inventory loss during cycling and is detrimental to the lithium-ion transport.^39^^,^^40^^,^^41^^,^^42^^,^^43^^,^^44^^,^^45^^,^^46^^,^^47^ However, these structures have been previously overlooked because they can be easily removed by the washing and drying sample preparation steps common in other characterization techniques. Cryo-EM is able to preserve the native state of these extended SEI structures by vitrifying the solid-liquid interfacial structures, thus allowing researchers to better probe the nanoscale features at the interface.

**Figure 6:**
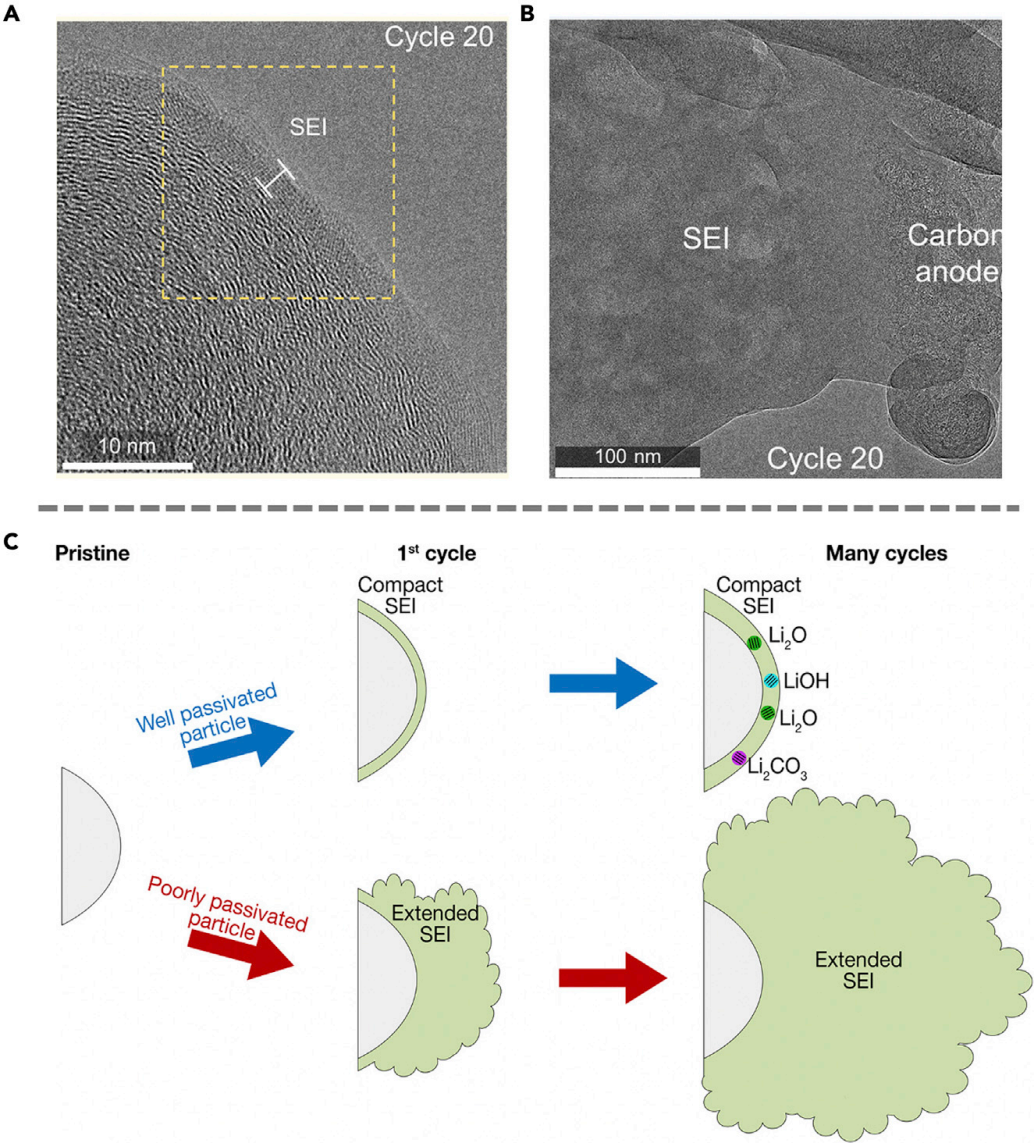
Extended SEI visualized under cryo-EM (A) Cryo-TEM images of late-cycle compact SEI showing direct interface with the carbon particle after 20 cycles. (B) Bright field cryo-TEM of large extended SEI deposits spanning hundreds of nanometers after 20 cycles. (C) Schematic of SEI formation on the carbon negative electrode. Adapted/reproduced from ref. ^39^, American Chemical Society.

A recent work studying the evolution of SEI growth on carbon black negative electrodes has directly demonstrated the formation of extended SEI.^39^ Using cryo-EM, the compact SEI with a thickness of ∼5 nm (Figures 6A and 6B) is observed to feature a high concentration of crystalline inorganic components such as Li_2_O, LiOH, and Li_2_CO_3_, which can effectively passivate the negative electrode due to their electronic properties such as low conductivity and high dielectric constant that mitigate electron transfer, as well as the high elastic modulus for better mechanical stability. As the battery cycles, the extended SEI grows along with the compact SEI, with the exception that the extended SEI extends to hundreds of nanometers and fails to provide sufficient passivation. The extended SEI is identified as a primary consumer of lithium ions and can result in an increase in electrode porosity, which in turn increases the overpotential for Li-ion transport and is detrimental to the late-cycle stability of the battery. Note that while the possibility of a large-scale and porous SEI layer has been previously discussed in some of the SEI growth models, cryo-EM allowed direct observation of this previously proposed SEI structure.^26^^,^^40^^,^^41^^,^^42^ Interestingly, instead of both layers forming on the same particle, the compact and extended SEI layers are observed on separate particles. Cryo-EM characterizations of both layers further indicate that the lack of inorganic crystalline SEI particles in extended SEI might be the reason for its poor passivation.

There are several hypotheses as to how an extended SEI forms.^39^ The first suggests that electrolyte molecules can transport through the relatively porous SEI that is lacking in inorganic components, resulting in continuous growth of extended SEI. Another plausible pathway is the reaction and precipitation of radicals generated during SEI nucleation. These radicals are products of the reduction of electrolyte molecules that might remain in the liquid phase and react in the electrolyte, the reaction products of which can precipitate on the negative electrode. The radicals may also propagate through the compact SEI that is lacking in crystalline SEI components. Overall, it can be generalized that well-passivated particles are expected to grow into a compact SEI while the poorly passivated particles tend to form extended SEI (Figure 6C).

**Figure 7:**
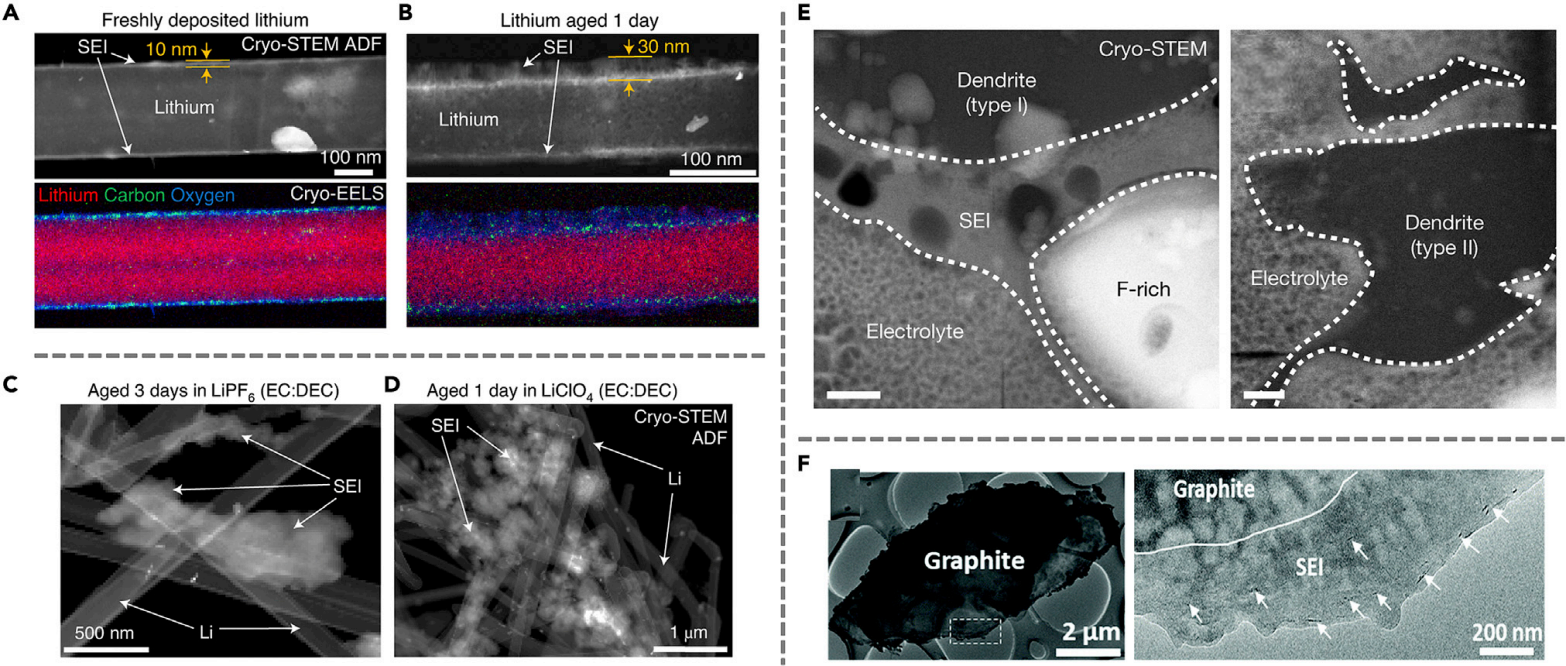
Various modes of extended SEI formation (A) The cryo-STEM ADF and cryo-EELS mapping of freshly deposited lithium with 10 nm of SEI. (B) Extended SEI formation upon aging the lithium for a day. (C) Cryo-STEM ADF images of Li anode aged in LiPF_6_ (EC:DEC). (D) Cryo-STEM ADF images of Li anode aged in LiClO_4_ (EC:DEC). (E) HAADF cryo-STEM imaging show the formation of an extended SEI layer on the type I dendrite but not on the type II dendrite. (F) The overview of the graphite upon cycling in 1M LiPF_6_ in EC:DEC after 200 cycles. SEI with a thickness of approximately 450 nm can be observed. Panels (A-D) are adapted/reproduced from ref. ^43^, Springer Nature Limited. Panel (E) is adapted/reproduced from ref. ^44^, Springer Nature Limited. Panel (F) is adapted/reproduced from ref. ^46^, Royal Society of Chemistry.

Calendar aging has recently been highlighted as the main contributor to capacity loss and degradation of Li metal batteries.^43^ To better understand the underlying mechanism of this corrosion phenomenon during aging, cryo-EM has been employed to characterize the SEI growth as the battery is at rest. In this regard, an increase in compact SEI thickness has been observed with battery aging in various electrolyte systems. Besides the typically observed compact SEI, extended SEI is also observed for Li metal aged for 24 h or longer (Figures 7A and 7B). Cryo-STEM electron energy loss spectroscopy (EELS) mapping results show a much stronger C-H signal in the extended SEI, suggesting that extended SEI features a large amount of soluble organic compounds such as carbonate-based polymers. While extended SEI is present in all electrolyte systems, the morphology and chemistry vary across the different electrolyte chemistries. For instance, the extended SEI in LiPF_6_ (EC:DEC) consists of dense clumps with high fluorine content. The LiF from the reduction of HF impurities precipitates outside of the compact SEI as “indirect” SEI, which will be discussed in greater detail in the next section. Changing the electrolyte salt to LiClO_4_, however, results in extended SEI consisting of large and porous clumps of particles with a length of up to several microns as shown in Figures 7C and 7D. Although it is still a challenge to predict the exact SEI morphology formed in different electrolyte systems, these studies point to several possible growth mechanisms of extended SEI. Traditional SEI models explain the growth of compact SEI, which is likely due to the insoluble decomposition products precipitating on the interface. The large cluster observed with extended SEI, however, suggests that the mechanism involves soluble decomposition products. The accumulation of precipitates formed by soluble radical species that propagate in the liquid phase and various decomposition products can increase the interfacial impedance, exacerbating capacity loss.

Cryo-EM has also provided insights into the correlation between Li dendrite morphology and SEI formation. A recent study by Kourkoutis et al. reported two types of dendrites with distinct SEI structures.^44^ While type I dendrites are smaller in scale (∼5 μm) and feature a 300-500 nm thick extended SEI layer, type II dendrites are hundreds of nanometers thick with only a thin layer of carbon-free and Li and oxygen-rich SEI layer. Although it is still unclear why extended SEI is not present on the type II dendrite, existing chemical mapping of the SEI layer suggests that it is primarily composed of lithium ethylene decarbonate species. This also explains the formation of large bubbles in the extended SEI, which is due to the production of ethylene gas during the process (Figure 7E).

Recently, extended SEI has also been observed in graphite anode systems using cryo-EM.^39^ In this case, SEI formation is closely associated with graphite exfoliation. Following the insertion of electrolyte molecules, an increase in the layer distance can be observed, which in turn disrupts the surface lattice and exfoliates the graphite layers. It is known that exfoliated graphene layers are electronically conductive,^45^ which results in a non-uniform electric field and electron tunneling effect. These layers can facilitate electron transport, allowing electrons to tunnel into the liquid region to exacerbate the decomposition of electrolyte species. Consequently, the exfoliated graphene layer-embedded SEI becomes unstable and continues to grow to a thickness of approximately 450 nm (Figure 7F). The incorporation of electrolyte additives such as ethylene sulfate (DTD), Triphenylphosphine (TPP), vinylene carbonate (VC), and fluorine-containing species, however, is found to be an effective way to mitigate the continuous growth of SEI due to graphite exfoliation. These additives work by having a high reduction potential and are preferentially reduced at the anode surface to form the desired SEI. The SEI formed by these species typically consists of compact crystalline inorganic compounds and is more stable in nature. This study demonstrates that the insights obtained from cryo-EM characterizations not only enable researchers to better understand the origin of instabilities but also develop strategies to mitigate them.

**Figure 8:**
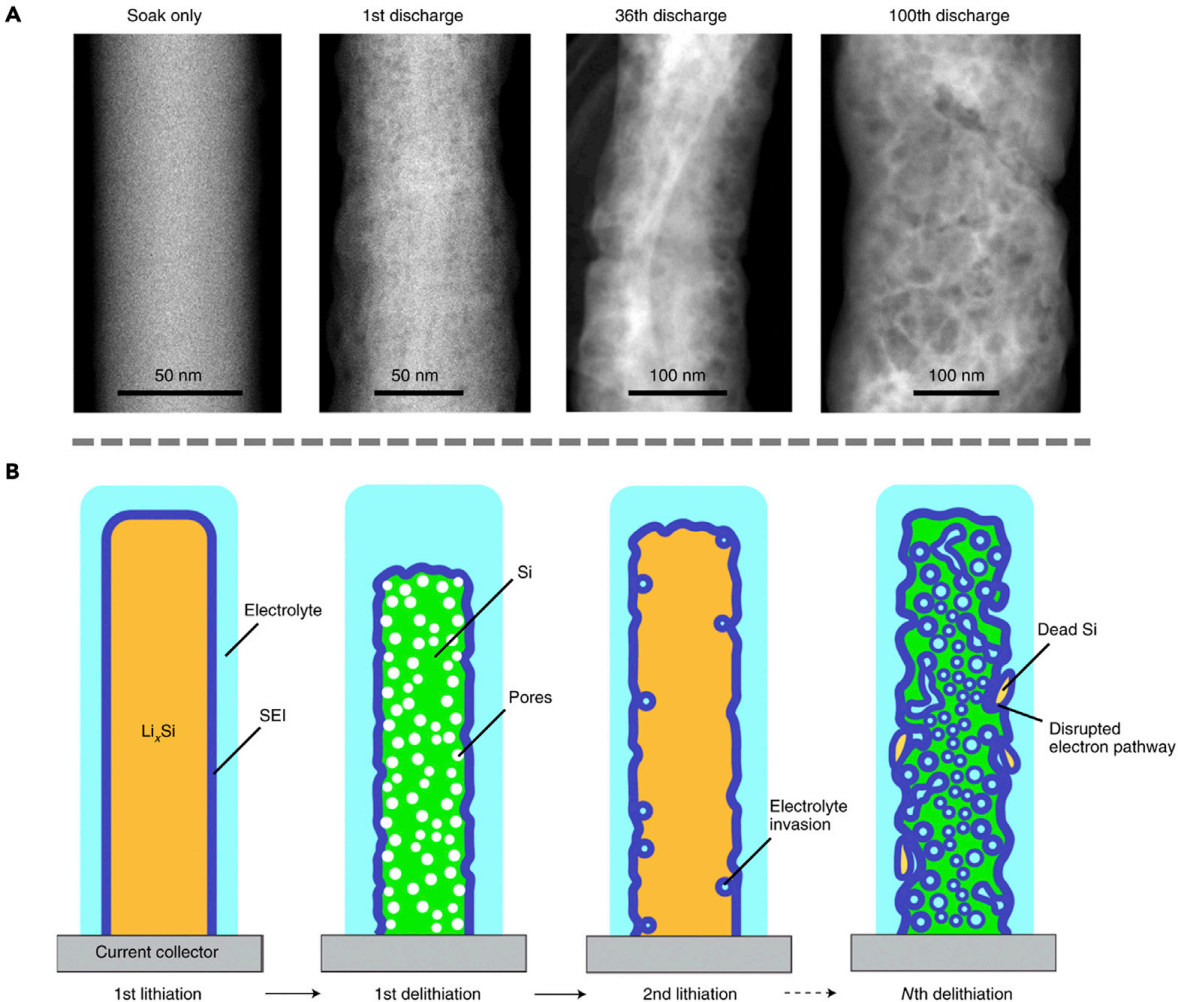
Inward growth of extended SEI observed under cryo-EM (A) Cryo-STEM-HAADF images of the Si nanowires at different cycle numbers. As the cell cycles, the nanowire becomes increasingly porous. (B) Schematic showing the porous structure of the nanowire upon continuous cycling. Adapted/reproduced from ref. ^47^, Springer Nature Limited.

The traditional definition of extended SEI as mentioned at the beginning of this section is an interface with hundreds of nanometers extending into the electrolyte. However, extended SEI is not only restricted to those that extend outward, but can also describe those extending inwards into the electrode material. One example is SEI grown on silicon (Si) nanowires.^47^ It is commonly known that Si nanowires tend to develop into more porous structures during cycling, a phenomenon that stems from vacancy condensation due to Li-ion extraction.^48^ As shown in the cryo-STEM-HAADF (high-angle annular dark-field) images (Figure 8A), the initial SEI that forms on Si consists of fluorine, oxygen, carbon, and phosphorous with a thickness of ∼20 nm. As the Si core grows increasingly porous, the SEI layer begins to extend into the nanowires, resulting in a “plum pudding”-like structure with a blend of Si and SEI. This is most likely due to the channels created in the Si nanowire as the internal pores increase in size and connect, facilitating the permeation of electrolyte species. Such gradual growth of extended SEI inwards into the Si nanowire directly leads to capacity loss as it disrupts conduction pathways within the Si core, which will eventually cause the Si domains to be isolated from each other as the extended SEI engulfs the electrode materials (Figure 8B). This study highlights the merits of cryo-EM, particularly its capability to directly observe extended SEI evolution and identify potential failure mechanisms.

### Indirect solid electrolyte interphase

**Figure 9:**
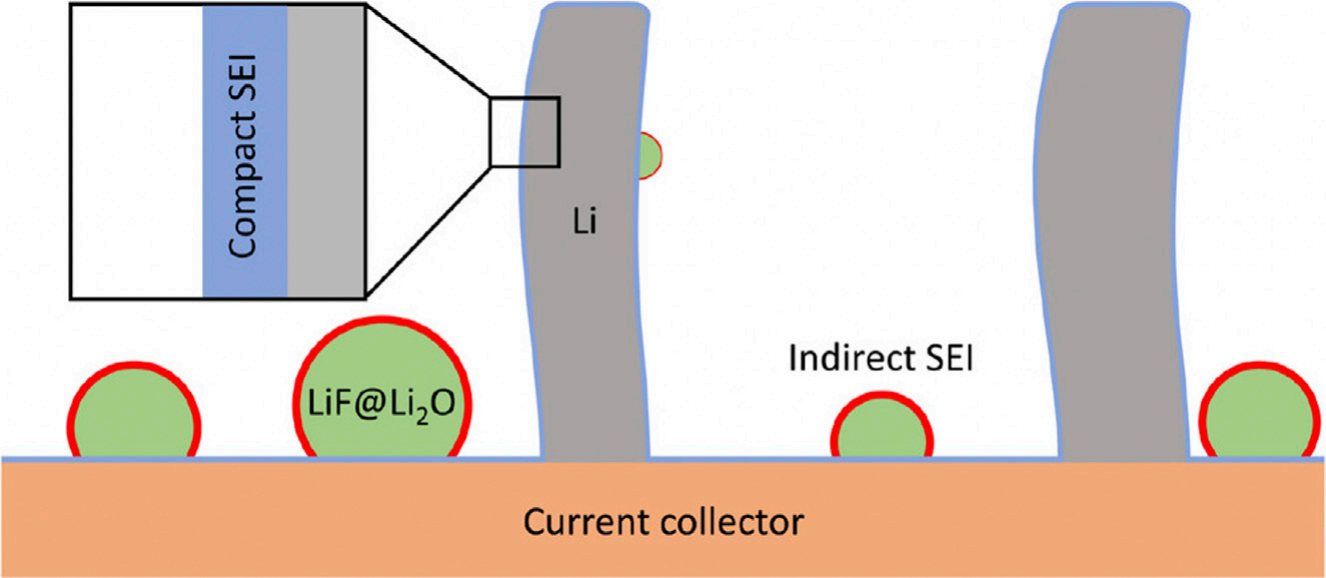
Schematic showing compact and indirect SEI Adapted/reproduced from ref. ^26^, American Chemical Society.

In comparison to the more well-understood compact SEI and extended SEI, the concept of an indirect SEI has only been identified very recently and studies on its properties are relatively sparse. In contrast to the compact and extended SEI that is interfaced directly on an anode surface, an indirect SEI describes components that are delocalized from the anode surface. The concept of an indirect SEI enriches our conventional understanding of SEI derived from ensemble-averaged characterizations and revises our understanding of the role of certain SEI components. The indirect SEI was first observed when elucidating the role of LiF in passivating the anode.^26^ The benefits of LiF have been demonstrated in a number of studies,^49^^,^^50^^,^^51^ and it is well-known for its excellent passivation properties and high electrochemical stability. Although it is commonly accepted that LiF, the decomposition product of fluorinated electrolytes, is favorable to battery cyclability, its significance in SEI formation is less understood. In this respect, cryo-EM is selected as the optimal tool to characterize the nanoscale distribution of SEI components in an effort to illuminate the role of LiF. Surprisingly, the results demonstrate that LiF is not actually present in the compact SEI directly interfaced with the anode surface, but extends outside of the compact SEI to form an indirect SEI layer (Figure 9). In comparison to Li_2_O, which is the dominating species in the inorganic layer of compact SEI because of its low solubility, LiF with higher solubility precipitates as nanoparticles with sparse coverage of the active material. Since it is not embedded in the compact SEI that passivates the active material, it is likely that LiF passivates the anode through a different mechanism than previously though. The presence of LiF, although not directly contributing to the compact SEI passivation film, may still promote a more uniform and less porous Li plating since it is also observed on current collectors. The presence of an indirect SEI demonstrates that not all SEI components are embedded in the compact or extended SEI. The study further highlights that the role of each SEI component should be extensively investigated as their decomposition mechanism may have important implications in optimizing the battery performance.

Overall, the application of cryo-EM in battery science over the past 5 years has facilitated tremendous advancements in our understanding of sensitive SEI surfaces. With the growing interest in cryo-EM for battery materials, fundamental understandings in the underlying mechanisms of SEI formation will continue to be enriched to enable next-generation high-performing battery systems.

### New cryogenic electron microscopy imaging modalities

There are a multitude of techniques that can facilitate the quantitative understanding of the SEI layer using transmission electron microscopy (TEM). We can break up the understanding of this interfacial layer into several components: understanding the atomic positions (diffraction/high-resolution imaging^52^), understanding electronic band structure (iDPC or integrated differential phase contrast, EBIC or electron beam induced contrast), understanding chemical bonds through phonon structure, and understanding transport using ultrafast TEM.

High-resolution imaging is only possible if the material can withstand a large dose of radiation imparted to it. Lithium metal, being a light atom, is susceptible to radiation damage. The momentum carried by the electron beam is sufficient to knockout the atomic nucleus from the lattice, and this process quickly (within seconds) destroys this material. Given lithium’s high conductivity, any radiolysis can be compensated for by supplying electrons from the electron microscope electrical ground.

Surprisingly, at cryogenic temperatures, lithium metal is resistant to radiation damage. The kinetics of the knock-on effect should not be influenced by temperature as the momentum transfer is much larger than the 25 meV of thermal energy the nucleus has at room temperature.^53^ However, Li et al.^15^ observed that as the temperature is lowered, the lithium metal survives longer and longer. At liquid nitrogen temperatures, no significant damage to the lithium metal has been observed. This ability to withstand radiation dose opens up a wide range of electron microscopy characterization techniques that would not otherwise be possible.

Imaging of the lithium lattice is the most direct technique. In both parallel beam illumination and focused probe scanning transmission electron microscopy, the lithium lattice remains intact and readily measurable out to between 1Å and 2Å resolution. The orientation of the lattice planes can be gleaned from the Fourier transform of the images, but with only a small sampling of lattice planes visible due to the lack of resolution, it is hard to determine the structure of any lithium compounds beyond very simple ones. The SEI layer has a patchy network of crystalline areas and is much more dose sensitive than lithium metal, making direct imaging a more difficult task to take in SEI identification.

The simplest solution to improve direct imaging is to have better detectors. For parallel beam TEM imaging, this means using direct electron detectors, and for STEM imaging this mean moving toward 4D-STEM techniques. Direct electron detectors improve the dynamic quantum efficiency over CMOS and CCD cameras, easily acquiring images at the information limit of the microscope (typically about 1Å). Being more efficient, these cameras also need fewer electrons to reach this limit.

In STEM mode, the electron probe can be either focused to a point for high-resolution lattice or atomic (≤1Å) resolution, or it can be slightly defocused to make a diffraction pattern in a small area. In the latter case, we can raster the defocused probe over the sample and acquire a diffraction pattern at each location. The latter case, 4D-STEM, is useful for point-to-point crystal structure information, as the observed reflections are not limited by electron wave coherence as they are in the case of direct imaging.

Tomography, tilting the sample to various angles and then stitching the images together to generate a 3D model, can be used with both direct imaging and 4D-STEM. In the former case a real space image of the SEI and lithium can be determined, although this is very difficult to do at atomic resolution, in particular in dose-sensitive materials.^54^ 4D-STEM techniques applied at different angles is analogous to 3D electron diffraction (or microED) sampling and is potentially very powerful if the sample is sufficiently crystalline.

Likewise, when the crystal structure is already known, then an analogous technique transmission Kikuchi diffraction (TKD) or transmission electron backscatter diffraction (tEBSD) can be used to determine the local crystal structure with resolution beyond what can be acquired with 4D-STEM. In this case, the transmitted electron beam diffracts not as spots, but as Kikuchi lines. These Kikuchi lines, formed as a multiple scattering effect between elastic and inelastic collisions in the material, can be used to determine the crystal structure by matching to known Kikuchi lines from various possible solutions. This could identify individual crystalline sections of the SEI and lithium metal without the need to tilt the sample, as is the case with 3D ED. In addition, only one scanning acquisition is required, although this technique is limited by the substation dose that is typically required.

Farther away from scattering with the nucleus, the probing electron scatters from the electrons in atomic orbitals. The incident electron loses energy in collisions with other electrons as they can transfer a significant amount of momentum to each other. These energy loss events can be measured using electron energy loss spectroscopy (EELS). The largest cross-section is from the interaction with the valence electrons in the material, often resulting in the creation of a bulk plasmon. These plasmons can be used to determine if pure lithium metal or some alloy is present, and can map these differences using a sub-10 nm size probe. Smaller cross-sections occur for K-shell and L-shell scattering events. The near-edge fine structure can be used to measure the local density of states and the chemical shifts as lithium changes its bonding environment when attached to different compounds. Although EELS has found its application in battery materials and yielded valuable insights, its full potential has yet to be exploited.^44^

Electrons can also lose energy when they interact with the nucleus, but the cross-section and energy loss are both small compared to almost all other cross-sections and energy loss events in EELS. A special spectrometer and monochromater are needed to see these 100 meV sized events, and the acquisition time makes mapping the changes in these signals small. The creation and destruction of phonons have not been applied to lithium batteries and the SEI for another reason. Microscopes with these high-resolution spectrometers are rare, and rarer still is their pairing with side entry stages that facilitate cryo-transfer holders.

We have discussed probing the nucleus, core-shell electrons, and valence electrons using spectroscopy. But there is another important attribute to consider measuring: local fields inside of the SEI and at interfaces. At the junction between two materials with different work functions, an electric field forms. Measuring this electric field is vital to understanding the impedance of both electrons and ions into and out of the SEI layer. Two techniques can be used to determine this field: iDPC and EBIC. The former uses a segmented detector and the effects of electric (and even magnetic) forces on the electron beam to determine small shifts to due fields in the valence band and field near the nucleus. These deflections are small and require a sensitive detector to measure. The latter, EBIC, places direct electrical connections on the sample and measures the separated electron-hole pairs on the opposite sides of the interface. This is a powerful technique, but sample preparation can be difficult.

The above highlights several under-leveraged techniques that can be combined with cryo-EM to enrich our understanding of SEI properties and their impact on battery performance. We anticipate these advances and others will be central to future efforts in broadening the scope of cryo-EM for battery research and beyond.

### Conclusions

The exploration of next-generation energy storage devices relies heavily on advanced characterization techniques. Motivated by the various limitations of current techniques, this perspective highlights the role of cryo-EM toward enhancing our understanding of the intricate interfaces formed during battery cycling.

Although much progress has been made in SEI research, there remain several major challenges that must be addressed to further develop a more comprehensive understanding of the SEI. The first of which is a closer investigation of the SEI composition. As discussed previously, the commonly used techniques such as XPS can provide surface information to a certain extent, but the low in-plane spatial resolution poses a challenge to clearly identifying the distribution. Additionally, while it is known that the composition of SEI is closely associated with the performance of lithium-based batteries, quantitative correlations and descriptors remain lacking.^10^ One example of which is the role of LiF, which has been believed to facilitate homogeneous Li^+^ flux and dendrite suppression.^55^ However, there is no clear mechanistic explanation to support this claim. In fact, recent cryogenic electron microscopy studies suggest that LiF does not contribute directly to the formation of a compact SEI passivation film, but rather precipitates to form an indirect SEI layer that is adjacent to and not in contact with the anode material. Therefore, not only is it necessary to observe the composition of SEI, advanced techniques that can provide insights into the correlation between the individual SEI components and battery performance is needed in future research. Another challenge of the SEI is monitoring its evolution process during cycling, which provides critical insights into the electrochemical reaction mechanisms. Yet, this cannot be achieved through *ex situ* characterization techniques due to high sensitivity of SEI in the ambient environment. Therefore, future studies should also aim to obtain real-time SEI evolution through advanced operando techniques and time-dependent cryo-EM studies.

In this regard, the high-resolution images captured by cryo-EM have offered new insights into the nanoscale structures and the underlying SEI formation mechanisms. Future studies on cryo-EM will undoubtedly grow in scale and impact, clarifying some of the most elusive concepts in battery science. For instance, cryo-EM can be implemented to examine some of the SEI phases (i.e. Li_2_O) with well-studied transport properties. Coupled with these controlled models of SEI films, cryo-EM can greatly enhance our understanding of the SEI.^56^ Additionally, considering that TEM is a local technique, heterogeneous structures such as the SEI can be captured by taking multiple images across different samples. One example of this is the visualization of dendrite growth in lithium metal batteries,^12^ where multiple images are taken along different growth directions (<111>, <211>, <110>) and statistical analysis is carried out to display representative results. The same strategy can be applied to cryo-EM-based SEI studies.

However, to reach the level of understanding that is desired for designing the most stable and efficient battery systems, several challenges must first be addressed based on the current cryo-EM technology. Firstly, increased accessibility and ease of characterization must be achieved for cryo-EM to develop into a people’s technique. In comparison to the other more broadly used characterization techniques such as SEM, the operation of cryo-EM is still rather complicated, and extensive training is required before researchers can fully master the technique. Another potential pitfall is that the images captured by cryo-EM are commonly assumed to be pristine and accurately reflect the structures of interest. This may not always be the case as improper control experiments, variations in sample protocols, and characterization of beam damage can all introduce errors to what is captured under cryo-EM. More standardized sample preparation protocols and well-documented procedures are thus necessary to guarantee the accuracy of the technique.
